# Comprehensive Review of Surgical and Radiological Management of Hemorrhagic Pancreatitis: Current Strategies and Outcomes

**DOI:** 10.7759/cureus.65064

**Published:** 2024-07-21

**Authors:** Camila Sanchez Cruz, Nathnael Abera Woldehana, Lorraine Ponce-Lujan, Pranay Shettywarangale, Pallavi Shekhawat, Naofal da Silva, Kevin A Reyes Gochi, Mario D Reyes Gochi

**Affiliations:** 1 General Practice, Universidad Nacional Autonoma de Mexico, Mexico City, MEX; 2 Public Health, Johns Hopkins Bloomberg School of Public Health, Baltimore, USA; 3 General Practice, Universidad de San Martin de Porres, Lima, PER; 4 General Practice, Kamineni Academy of Medical Sciences and Research Centre, Hyderabad, IND; 5 Obstetrics and Gynaecology, Postgraduate Institute of Medical Sciences and Research (PGIMSR) and Employees' State Insurance (ESI) Model Hospital, Delhi, IND; 6 Surgery, AdventHealth Tampa, Tampa, USA; 7 Faculty of Medicine, Universidad Nacional Autónoma de México, Mexico City, MEX; 8 General Practice, Universidad Nacional Autónoma de México, Mexico City, MEX

**Keywords:** pancreatitis, vascular disruptions, interventional radiology techniques, diagnostic imaging, hemorrhagic pancreatitis

## Abstract

Hemorrhagic pancreatitis, a severe complication of acute and chronic pancreatitis, involves bleeding due to vascular disruptions. This condition presents significant clinical challenges and is associated with high morbidity and mortality. The bleeding can result from arterial or venous complications, often exacerbated by inflammatory and enzymatic damage to blood vessels within the pancreas. Patients with hemorrhagic pancreatitis may experience symptoms such as abdominal pain, nausea, vomiting, and gastrointestinal bleeding. Diagnostic imaging, including CT and MRI, is crucial in identifying the source of bleeding and guiding treatment decisions. Management strategies have evolved over the past two decades, shifting from purely surgical approaches to including interventional radiology techniques. Surgical intervention is often reserved for hemodynamically unstable patients or those with large pseudoaneurysms, offering definitive treatment but carrying higher risks of complications. Endovascular techniques, such as transcatheter embolization, provide a less invasive alternative with high success rates and shorter recovery times, though rebleeding may occur. Treatment choice depends on various factors, including the patient's stability, the size and location of the bleeding, and the availability of specialized expertise. Overall, the management of hemorrhagic pancreatitis requires a multidisciplinary approach, combining surgical and radiological techniques to optimize patient outcomes and reduce the risk of mortality. Long-term follow-up is essential to monitor for recurrent disease and manage the metabolic consequences of pancreatic insufficiency.

## Introduction and background

Pancreatitis is an inflammatory disorder of the pancreas, and bleeding is considered a rare but severe complication of acute and chronic pancreatitis [1,2]. Two types of pancreatitis are described: interstitial and necrotic. Interstitial pancreatitis is reported to be associated with bleeding in 1.5% of patients, while necrotizing pancreatitis increases the percentage to 13.5% [1].

According to the revision of the Atlanta Classification of Pancreatitis, acute pancreatitis can have two phases: an early and a late phase. The early phase lasts approximately one week, followed by the late phase, which can last weeks to months. Mild cases of pancreatitis usually happen during the early phase, although there are cases where patients may have transient (<48 hours) or persistent (>48 hours) organ failure. The late phase shows the persistence of systemic signs of inflammation or local complications; therefore, the late phase occurs only in patients with moderately severe or severe pancreatitis. In these patients, local complications such as necrosis, thrombosis of splenic and portal veins, and peripancreatic collections could later cause hemorrhage or necrosis, leading to single or multiorgan damage [3-5]. Although it is not vastly studied, several publications suggest that intra-abdominal hemorrhage of a major vessel is considered the primary cause of more than half of deaths in patients affected by hemorrhagic pancreatitis, resulting in the worst outcomes [1,2,6].

Vascular complications are uncommon but could occur in 1-23% of patients with pancreatitis. Venous thrombotic complications are more common than arterial complications, with thrombosis of the portal and splenic veins occurring in up to 23% of these cases. Arterial complications are usually hemorrhagic and account for 1.3-10% of the vascular complications. The mortality of hemorrhage due to arterial vascular complications is 34-52%. Therefore, prompt management is required [2,7]. In the past two decades, hemorrhagic pancreatitis management has shifted between surgical and interventional radiology approaches [2]. This review aims to evaluate and compare the management strategies and outcomes.

## Review

Epidemiology and etiology****

The reported incidence of hemorrhagic pancreatitis is about 1-23%. It is often fatal in 34-50% of the cases [2,8]. Arterial complications account for 1-13% of all pancreatitis cases. Acute bleeding in pancreatitis is most commonly seen in cases of ruptured pseudoaneurysm, followed by portal vein hemorrhagic pseudocyst without pseudoaneurysms and capillary venous or minor vessel bleeding, at 60%, 20%, and 20%, respectively. Venous complications account for 1-23%; portal vein thrombosis affects 23% of patients, splenic vein thrombosis affects 22%, and superior mesenteric vein thrombosis affects 19% of patients with pancreatitis [2].

Demographically, hemorrhagic pancreatitis can affect individuals at any age but is more common in middle-aged adults [9]. Men are more frequently affected than women, primarily due to higher rates of alcohol consumption [10]. Chronic heavy alcohol use is a significant risk factor for acute pancreatitis, accounting for up to 40% of cases, with a higher likelihood of progressing to hemorrhagic pancreatitis [11]. Repeated episodes of acute pancreatitis or chronic conditions like alcoholism lead to chronic inflammation, damage, and fibrosis of pancreatic tissue, thereby increasing the risk of vascular complications and bleeding [12]. There are no significant racial or ethnic differences reported in the incidence of hemorrhagic pancreatitis. However, variations in underlying risk factors can influence occurrence rates [13]. Less common etiologies include trauma, infections, hyperlipidemia, and certain medications. Idiopathic cases, where no cause is identified, also occur [12].

Trauma, whether abdominal or iatrogenic, such as during the ERCP procedure, can directly damage pancreatic tissue and blood vessels, leading to bleeding. Infections, particularly bacterial translocation from the gut, often exacerbate pancreatic necrosis and may precipitate hemorrhagic complications by inducing a severe inflammatory response and enzymatic destruction of blood vessels within the pancreas [12,13]. These factors collectively contribute to the severe morbidity and potential mortality associated with hemorrhagic pancreatitis.

Pathophysiology****

Hemorrhage in pancreatitis can occur in the gastrointestinal tract, peritoneal cavity, or retroperitoneum and arise due to arterial or venous complications [14]. Arterial complications are caused by arterial wall disruption, weakening, and erosion from the action of free lipolytic and proteolytic enzymes in the retroperitoneum. The marked inflammatory response, necrosis, and release of pancreatic fluid with activated enzymes through different cellular signaling pathways can lead to the formation of a pseudoaneurysm, which may bleed or spontaneously rupture [2,6,15,16].

In the case of venous complications, such as venous thrombosis of the splenic-portal-mesenteric veins, bleeding can occur through esophagogastric varices [8,14,17]. Another complication that can lead to bleeding is the formation of pseudocysts. A pseudocyst is a localized peripancreatic fluid collection rich in pancreatic enzymes and surrounded by a wall of fibrous tissue with no epithelial lining, usually caused by the disruption of the pancreatic duct [18,19].

**Figure 1:**
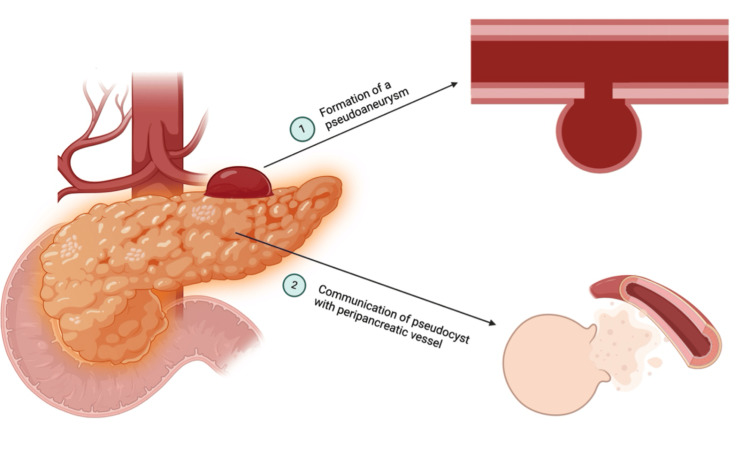
Mechanisms for the formation of pancreatic pseudoaneurysm 1. Severe inflammation, present in pancreatitis and pancreatic enzyme autodigestion, weakens the artery wall, forming a pseudoaneurysm. 2. Communication between an already-formed pseudocyst in the pancreas and a peripancreatic artery converts the pseudocyst into a large pseudoaneurysm [18-21]. Figure created with BioRender, all credits to Lorraine Ponce-Lujan.

Although hemorrhagic pseudocysts without pseudoaneurysms account for 20% of all acute hemorrhages in pancreatitis, bleeding from a ruptured pseudoaneurysm accounts for more than 50% of cases [2]. The role of pseudocysts in the formation of a pseudoaneurysm will be discussed as follows. An arterial pseudoaneurysm is a vessel that has ruptured and has a surrounding hematoma in communication with the vessel lumen. It results from injury to the vessel caused by surrounding inflammation and pancreatic enzymes [15]. As previously mentioned, the most common site of a pseudoaneurysm is the splenic artery, in almost 50% of cases. The percentage decreases to 20-25% for the gastroduodenal and pancreaticoduodenal vessels. Less common sites include the hepatic, mesenteric, and colic arteries [2,8,20]. Detection typically occurs 3-5 weeks following the onset of acute pancreatitis, but pseudoaneurysm bleeding can occur from a few days to several years after pancreatitis [2,20]. Two possible mechanisms account for the formation of pancreatic pseudoaneurysms. One involves severe inflammation and/or pancreatic enzyme autodigestion, with trypsin being the most important enzyme, causing pancreatic or peripancreatic vessel disruption with subsequent pseudoaneurysm formation. This typically develops as a complication following chronic or necrotizing pancreatitis, which is a life-threatening condition [18,19,21]. The other mechanism involves the communication of a pseudocyst with a peripancreatic vessel, converting the pseudocyst into a large pseudoaneurysm [19,20] (Figure 1).

Regarding other complications of pancreatitis, pancreatic or peripancreatic necrosis can develop in up to 72% of cases between two to four weeks from disease onset and is believed to be caused by the translocation of bacterial microorganisms from the intestinal lumen [22,23]. Other complications can include infection of a pancreatic pseudocyst, an enclosed collection of pancreatic secretions [24,25]. Additionally, there has been mention of infection of thrombosed pseudoaneurysms and extrinsic compression, causing a mass effect and necessitating drainage [2,21,26].

Diagnosis****

The more common symptoms at admission for pancreatitis include abdominal pain, nausea, vomiting, melena, fainting, and loss of appetite. There are also reports of asymptomatic cases, cholestasis, and gastrointestinal bleeding [27]. In some cases, gastroesophageal varices, gastroduodenal, and superior pancreaticoduodenal vessels are the sources of bleeding, thus causing gastrointestinal bleeding [28]. Additionally, there are reports of colonic bleeding associated with a mortality rate of 87.5% in acute and 38.8% in chronic pancreatitis [28]. Clinical signs such as Fox's sign, Cullen's sign, and Grey Turner's sign have been reported in these patients [29,30]. Similar laboratory patterns as acute pancreatitis can be found, such as elevation of amylase and lipase levels. Because of the potential for peritoneal hemorrhage and reduced hemoglobin levels, the hemoglobin trend should be monitored [8]. It is important to first consider common causes of upper gastrointestinal bleeding (e.g., gastroduodenal ulcers, gastroesophageal varices) within the context of chronic alcohol use, which is typically more common [28].

Diagnostic Imaging

Most cases of upper and lower endoscopy only exclude primary gastrointestinal bleeding but do not confirm the diagnosis. The gold standard technique is surgical exploratory laparotomy, which plays an important role in diagnosis and bleeding control [28]. Preoperative angiography functions as a diagnostic and therapeutic method in certain cases. Nevertheless, it has several limitations in detecting venous, intermittent arterial bleeding, and bleeding from a large surface area [28]. CT imaging may show the formation of arterial pseudoaneurysms, evidence of bleeding into a pancreatic pseudocyst or fluid collection, or portal venous thrombosis with the development of varices [31]. MRI findings of hemorrhagic lesions show spotted or patchy high signal intensity on T1-weighted images with fat suppression. Peripancreatic hemorrhage might be secondary to necrosis of peripancreatic tissue, visualized as patchy or large diffuse areas with hyperintensity on T1-weighted images [32]. Doppler ultrasound is useful in establishing a diagnosis and can determine the presence of flow in a pseudoaneurysm or the absence of flow due to portal venous thrombosis [31]. For all these imaging studies, it is important to consider the limitations and advantages of each one when deciding which to use. It is also crucial to note that the interpretation and use of these techniques require adequate training of the medical staff [33-35].

Surgical management

Surgical intervention remains crucial in managing severe hemorrhagic complications of pancreatitis despite the increasing use of endovascular techniques. Primary indications include hemodynamic instability requiring immediate bleeding control, failed angiographic embolization, and large pseudoaneurysms (typically >10 cm) at risk of rupture or infection [26]. Other indications include infected pancreatic necrosis, persistent organ failure, and extrinsic compression of surrounding structures [36]. The timing of surgery is critical, with a trend towards delayed intervention (ideally > four weeks after the onset of pancreatitis) to allow better differentiation of necrotic tissue and reduced surgical morbidity [37]. However, emergency surgery may be necessary for uncontrolled bleeding or rapid clinical deterioration [26].

Delayed intervention allows for better differentiation between viable and nonviable tissue, reducing the extent of necrosectomy required and thus decreasing the risk of postoperative complications [38]. Infected pancreatic necrosis often necessitates surgical intervention, as it is associated with high mortality rates if left untreated. Persistent organ failure, particularly involving the cardiovascular, respiratory, or renal systems, may also require surgical management to prevent further deterioration [39]. Extrinsic compression of surrounding structures, such as the bile ducts or gastrointestinal tract, by inflammatory masses or pseudocysts can lead to obstructive symptoms that necessitate surgical relief [40].

Surgical Techniques

Many surgical approaches have been described, ranging from pancreatectomy (of varying extent) to ligation of the culprit vessels. As pseudoaneurysms constitute the majority (69%) of underlying causes of bleeding in chronic pancreatitis, surgical treatment often requires resection of these lesions [41].

Partial Pancreatectomy

Distal pancreatectomy, with or without splenectomy, is often performed for bleeding originating from the pancreatic tail or body [38]. The procedure involves resecting the affected portion of the pancreas and the source of bleeding. Mortality rates for this procedure in hemorrhagic pancreatitis range from 0 to 23%-25%. The mortality rates, blood loss, and transfusion needs have not been significantly modified during pancreatectomy despite the use of concomitant tranexamic acid, which has been proven effective in other pathologies. This highlights the complicated management of these patients [42-44]. Postoperative pancreatic fistula remains a significant concern, occurring in up to 30% of cases [39]. Advances in surgical techniques and perioperative care have improved outcomes, although the risk of pancreatic fistula formation remains a major challenge [45].

Resection of Pseudoaneurysms

Surgical resection of pancreatic pseudoaneurysms is indicated when endovascular management fails or is not feasible, particularly for large pseudoaneurysms [26]. The procedure involves careful dissection and removal of the pseudoaneurysm, often requiring concurrent management of the associated pancreatic pseudocyst. Mortality rates for elective resection are below 5% in experienced centers, but emergency resection carries significantly higher risks [40]. The complexity of the procedure demands high surgical expertise, and outcomes are significantly better in high-volume centers specializing in pancreatic surgery [46].

Ligation of Bleeding Vessels

Direct ligation of bleeding vessels is often necessary in emergent situations or when the bleeding source is identified intraoperatively [26]. This technique is particularly useful for controlling bleeding from the gastroduodenal or splenic arteries. Reported mortality rates for emergency ligation procedures range from 15% to 40%, reflecting the critical condition of these patients [26]. Intraoperative identification of the bleeding source is crucial, and preoperative imaging can aid in planning the surgical approach [47].

Postoperative Complications

Surgical management of hemorrhagic pancreatitis is associated with significant postoperative morbidity. Common complications include pancreatic fistula formation (10-30%), intra-abdominal abscess (15-25%), and wound infections (10-20%) [45]. More severe complications, such as postoperative bleeding (5-10%) and multiorgan failure (10-20%), can occur, particularly in patients with preexisting organ dysfunction [37]. One study demonstrated that rebleeding rates were higher in patients who underwent ligation of the bleeding vessel compared to those who had partial pancreatectomy [41]. Management of these complications often requires a multidisciplinary approach. Pancreatic fistulas may necessitate prolonged drainage or repeat interventions, while intra-abdominal abscesses frequently require percutaneous drainage. Postoperative hemorrhage might necessitate urgent repeat angiography or reoperation [37].

Proactive postoperative management includes vigilant monitoring for signs of complications, early intervention when they occur, and supportive care tailored to the patient's needs. Nutritional support, infection control, and management of organ dysfunction are integral components of postoperative care, as these patients can present with higher rates of infections or nutritional deficiencies [48,49]. Implementing enhanced recovery after surgery (ERAS) protocols has shown promise in reducing postoperative complications and improving overall outcomes in patients undergoing major pancreatic surgery [50].

Long-Term Outcomes

The long-term prognosis for patients undergoing surgery for hemorrhagic pancreatitis varies widely depending on the severity of the initial disease and the extent of pancreatic resection. Studies report 5-year survival rates ranging from 50-70% [26]. Many patients experience some degree of pancreatic insufficiency, with 20-40% developing diabetes mellitus and 30-50% requiring pancreatic enzyme supplementation [39]. Follow-up typically involves regular clinic visits to monitor for recurrence of pancreatitis or pseudocyst formation. Imaging studies such as CT or MRI are often performed at three- to six-month intervals initially, then annually. Patients require lifelong pancreatic function monitoring, including periodic endocrine and exocrine assessments [39].

Long-term management includes addressing the metabolic consequences of pancreatic surgery. Patients with diabetes mellitus require ongoing endocrinological support, and those with exocrine insufficiency need pancreatic enzyme replacement therapy. Regular follow-up is crucial for early detection of recurrent disease or new complications. Lifelong surveillance helps optimize patient outcomes and quality of life [51].

Radiological Management

Timely intervention is warranted when there is a pancreatitis hemorrhage suggested by CT evidence of the presence of hematomas, hemorrhagic pseudocysts, extravasation of contrast media, or the formation of arterial pseudoaneurysm, which can be either surgical or radiological, especially for active bleeding and pseudoaneurysm formation [52]. Although arterial pseudoaneurysms are sometimes clinically silent, the risk of rupture is high, accounting for 1 to 10 percent of arterial complications and 60 percent of all acute hemorrhages in pancreatitis [2,53]. Transcatheter embolization is preferred for hemodynamically stable patients, while surgery is the gold standard for managing ruptured pseudoaneurysms in hemodynamically unstable patients. However, when severe pancreatic inflammation makes surgery technically difficult, the endovascular approach becomes a better option, with resuscitative efforts preceding transcatheter embolization in unstable patients [54,55].

Selective Arterial Embolization

The mainstay of endovascular treatment is usually therapeutic transcatheter angiographic embolization, facilitated by diagnostic angiography [56,57]. The right common femoral approach is commonly used to catheterize the celiac and superior mesenteric artery for digital subtraction angiography. The images are analyzed to detect the extravasation of contrast medium, pseudoaneurysm formation, and other vascular abnormalities, then compared with initial CT results to confirm their validity [52]. The splenic artery (37.7%) is the most common location of pseudoaneurysm, followed by the gastroduodenal artery (23.6%), pancreaticoduodenal artery (10.6%), hepatic artery (8.3%), left gastric artery (3.3%), superior mesenteric artery (3.3%), and colic artery (2%) [58].

**Figure 2:**
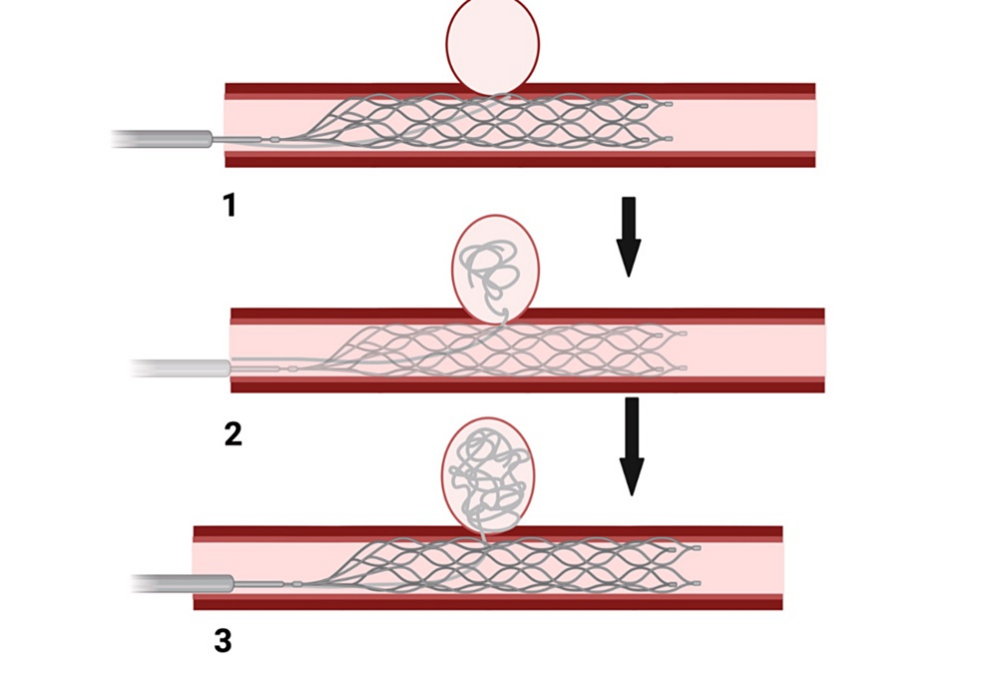
Stent graft expansion and coil deployment 1. A catheter was inserted via a transarterial approach and navigated to the aneurysm site. A collapsed stent graft is positioned at the target site and is expanded. 2. A coil is positioned at the target site and is deployed into the pseudoaneurysm. 3. The coil packs the pseudoaneurysm, blocking the blood flow to the pseudoaneurysm and excluding it from circulation [60,61]. Figure created with BioRender, all credits to Pranay Shettywarangale.

Platinum coils (Tornado or Nester, Cook Medical) and N-butyl cyanoacrylate (NBCA, Histoacryl, B. Braun Melsungen AG), combined with microparticles such as gelatin sponge, are the most commonly used embolic materials [59]. The choice of embolic material varies depending on many factors, including the artery involved, characteristics of the pseudoaneurysm including origin, size of the sac and neck, patient’s clinical status, operator preference and experience, availability, and cost. A single agent or a combination of two or more agents is often used. Coils have been reported to be safe and reliable, offering excellent control during deployment. Due to the rich collateral supply, coils can be safely deployed in abdominal vessels without significant risk for end-organ ischemia. Even major arteries like the splenic artery, hepatic artery, and gastroduodenal artery can be sacrificed without significant ischemia [60,61]. A normal coagulation profile is needed when coils are used for effective thrombosis [58] (Figure 2).

The principle of embolization is to block the blood flow from the bleeding site, including the pseudoaneurysm, both before and after the lesion to prevent bleeding due to backflow from collaterals [52]. The principle of pseudoaneurysm embolization involves excluding it from the arterial circulation, often achieved by microcoil placement from the distal to proximal ends of the pseudoaneurysm [62]. When bleeding was identified in one or more small distal branches, NBCA or gelatin sponge particles were administered via a microcatheter following precise catheterization. Using stent grafts to exclude arterial rupture or pseudoaneurysm has become increasingly popular, as it maintains blood flow to distal organs and prevents end-organ ischemia. Digital subtraction angiography (DSA) can be repeated post-procedure in both embolization and stent graft placements to verify that the vascular abnormality has been completely excluded [52,59].

Management of venous bleeding is more complicated than arterial bleeding owing to the difficulty in identifying and controlling the bleeding through angiographic procedures and the severity of localized tissue destruction [47].

Additional Imaging-Guided Therapy

Percutaneous thrombin injection into the pseudoaneurysm sac induces clotting and is another treatment option. The commercial product is a sterile protein solution with human fibrinogen and synthetic aprotinin mixed with human thrombin and calcium. Once injected, it forms a bio-absorbable clot, reducing the risks of infection, necrosis, and inflammation. Typically, a 1000-1500 IU dosage of human thrombin is preferred over bovine thrombin due to its lower anaphylactic risk. This procedure is performed under the guidance of CT or ultrasound (USG), which offers real-time visualization to minimize the risk of accidental punctures [63]. If cannulation or identification of the feeding vessel of the peripancreatic pseudoaneurysm fails, transabdominal thrombin injection can be the primary treatment, especially for surgically unfit patients. It also stabilizes the vessel, serving as a bridge to later surgical intervention [64].

Efficacy and Long-Term Outcomes

The assessment of successful hemostasis was based on several criteria: the absence of contrast media extravasation or pseudoaneurysm on the final angiogram, symptom resolution, stable lab results, vital signs, no recurrence or procedure-related complications, and follow-up CT scans showing resolved hemorrhage or pseudoaneurysm. The Society of Interventional Radiology guidelines classified complications as major or minor. The success rate in patients with acute and chronic pancreatitis is similar [58]. Rebleeding was managed with reintervention. Splenic artery embolization had higher risks of focal infarction and abscess [52], with rare complications like microcoil migration, subcapsular hematoma, radiodermatitis, and puncture site hematoma. Long-term follow-up showed good outcomes with rare recurrences, usually within six months [62]. Surgery and endovascular embolization serve complementary roles. Surgery is typically reserved for unstable patients, failed embolizations, or cases where embolization is deemed unsafe, and it is rarely needed for post-embolization complications.

Comparison of surgical and radiological management 

Surgical management offers direct access to the bleeding site and allows for definitive treatment, particularly in cases of large pseudoaneurysms or when endovascular approaches fail [26,39]. It is essential for hemodynamically unstable patients and can address concurrent issues such as infected necrosis [36]. Surgery provides the opportunity for comprehensive exploration, debridement, and drainage, which can be crucial in severe cases [37]. However, the invasive nature of surgical intervention can lead to prolonged recovery times and an increased risk of complications such as pancreatic fistula formation [26,36,37,39,47].

Radiological interventions, primarily endovascular embolization, are less invasive and can be performed on hemodynamically stable patients [52]. They offer quicker recovery times and the possibility of being repeated if necessary [39,52]. Endovascular techniques have evolved significantly, allowing for precise localization and treatment of bleeding sites with minimal collateral damage [47]. However, they may not be suitable for all cases, particularly large pseudoaneurysms or when the bleeding vessel cannot be safely catheterized [39]. The risk of rebleeding and the potential need for repeated interventions are important considerations [62].

Factors Influencing Treatment Choice

The choice between surgical and radiological management depends on several factors: hemodynamic stability of the patient, with unstable patients often requiring immediate surgical intervention [26,39]; size and location of the pseudoaneurysm, with larger pseudoaneurysms (>10 cm) potentially more suitable for surgical management [26,39]; presence of concurrent complications (e.g., infected necrosis), where surgery may be preferred to address multiple issues simultaneously [36]; availability of interventional radiology expertise, as centers with experienced interventional radiologists may favor endovascular approaches [39,52]; timing since the onset of pancreatitis, with a preference for delayed surgical intervention when possible, ideally more than four weeks after onset, to allow for better differentiation of necrotic tissue [36,37]; and the patient's overall condition and comorbidities, which can influence the ability to tolerate surgery or prolonged anesthesia [36].

Comparison of Success Rates and Complications

Endovascular embolization has shown high technical success rates, ranging from 75% to 100% [52]. This approach offers the advantage of precise localization and treatment of bleeding sites. However, rebleeding rates of 17-37% have been reported [62], necessitating vigilant follow-up and potential reintervention.

While more invasive, surgical management can provide definitive treatment with success rates of 70-85% [26,39]. It allows for direct visualization and control of the bleeding source and management of associated pancreatic pathology. However, it carries higher risks of complications, including pancreatic fistula (10-30%), intra-abdominal abscess (15-25%), and wound infections (10-20%) [26,36,39].

Mortality rates for emergency surgical interventions range from 15-40% [39,47], reflecting the critical condition of patients requiring such interventions. In contrast, mortality rates for endovascular procedures are generally lower, ranging from 0-14% [52]. However, these figures may reflect selection bias, as more stable patients are often chosen for endovascular procedures [62]. The lower mortality rates in endovascular interventions should be interpreted cautiously, considering the potential need for subsequent interventions or surgery in some cases. This information is summarized in Table 1.

**Table 1:** Comparison of surgical and radiological techniques [36,39,47]

Aspect	Surgical Techniques	Radiological Techniques
Success rate	70–89%	75–100%
Mortality rate	0–40%	0–20%
Rebleeding rate	12–50%	4–37%
Major complications	Pancreatic fistula, intra-abdominal abscess, wound infections	Splenic infarction, coil migration, access site complications
Advantages	Definitive treatment can address underlying pancreatic pathology	Less invasive, quicker recovery, can be repeated if necessary
Disadvantages	Higher morbidity, especially in the acute phase, is technically challenging due to inflammation	It may not be suitable for all cases, with the potential for rebleeding
Best for	Hemodynamically unstable patients, failed embolization, large pseudoaneurysms (>10 cm)	Stable patients, smaller pseudoaneurysms, high surgical-risk patients

Further research on surgical and radiological management in hemorrhagic pancreatitis should explore the comparative efficacy, long-term outcomes, and cost-effectiveness of these approaches. Additionally, investigations should focus on identifying patient-specific factors influencing treatment success, optimizing multidisciplinary care strategies, and developing guidelines for personalized management to improve patient prognosis and reduce morbidity and mortality [65].

## Conclusions

The management of hemorrhagic pancreatitis, characterized by severe complications such as arterial and venous bleeding, requires a multifaceted approach combining surgical and radiological interventions. Hemorrhagic complications, though rare, are associated with high mortality, underscoring the importance of prompt and effective treatment. The choice of management strategy hinges on various factors, including the patient's hemodynamic stability, the size and location of the pseudoaneurysm, the presence of concurrent complications, and the expertise available at the treating center. While surgical approaches are crucial for managing large or complex pseudoaneurysms and concurrent pathologies, radiological interventions serve as a valuable tool for stabilizing patients and controlling bleeding with minimal invasiveness. Integrating surgical and radiological techniques, guided by patient-specific factors and clinical judgment, can optimize outcomes in hemorrhagic pancreatitis.
